# Opening of the Hospital for Epilepsy and Paralysis

**Published:** 1903-06-20

**Authors:** 


### OPENING OF THE HOSPITAL FOR EPILEPSY AND PARALYSIS.

THE weather on the occasion of the opening of this hospital by Princess Louise, Duchess of Argyll, on Saturday, June 13th, was not a whit more favourable than it had been two days previously when the King and Queen visited the London Hospital. But the friends and supporters of the hospital were not deterred by the pouring rain, and mustered in large numbers in the ward on the first floor of the building where the opening ceremony took place. Outside the hospital a guard of honour, drawn from the 5th Middlesex Rifle Volunteers, with their band, was stationed. Her Royal Highness, who was accompanied by the Duke of Argyll and attended by Lady Sophia Macnamara, proceeded at once to the ward where the assembly were awaiting the arrival of the royal party.

The Earl of Hardwicke, President of the hospital, drew a brief sketch of its history from its foundation in 1866, when it was decided by a few pioneers, both doctors and laymen, to establish a hospital for the treatment of nervous diseases. A small house was taken in Marylebone to begin with, but this was outgrown in 1872, and they acquired Winterton House in Regent's Park, where they remained till 1900, when the lease terminated and was not renewable. The authorities were then faced with the alternative of either abandoning the work of the charity altogether or acquiring new premises. Fortunately, by legacies and donations they were helped out of their difficulty and were enabled to erect the new hospital.

Mr. G. A. Knapp-Fisher, member of the Committee of Management, said that the completed hospital would afford accommodation for 60 beds and a large number of out-patients. The present building contained 40 beds and they proposed to start work at once and leave future erection to the "dead hand," but it would have to be supported by the "living hand." They had many great expenses, amongst which the item of rates was very heavy and more than absorbed their normal income. They considered that, being situated in the wealthy borough of Marylebone and on the confines of the borough of Paddington, they might look to these neighbours to support them, not only with money but with gifts of many kinds. The hospital was still very bare; they would like pictures, but the doctors considered they conveyed microbes and so must be avoided. Another wing would in due course be erected at a probable cost of £11,000 or £12,000, including fitting and furnishing. The whole cost of the hospital would then amount to £40,000. In conclusion Mr. Knapp-Fisher paid a hearty tribute of thanks to the services rendered by the secretary and the matron.

Princess Louise then said in a clear voice, "It gives me much pleasure to be here to-day and to declare this hospital now open."

A short service of dedication was conducted by the Bishop of London, who attended in his robes.

Canon Duckworth then moved a vote of thanks to Her Royal Highness for her great kindness in performing the opening ceremony, which was seconded by Dr. George Ogilvie, senior member of the acting medical staff. Dr. Ogilvie, in the course of his remarks, expressed the hope that the hospital had entered upon a new career of usefulness, and that Her Royal Highness's visit there that day would do much to help them in their endeavours. The hospital was not a general nor a local one but was a special hospital for the treatment of diseases of the nervous system, and their patients came not only from all parts of London and the suburbs, but from the provinces. The staff of the hospital were thus afforded great opportunities for the study of every form of nervous disease.

The Duke of Argyll, who acknowledged the vote of thanks on behalf of the Princess, said that they need not fear the lack of adequate support for the hospital on the ground of its being small. They heard a great deal nowadays about magnificent hospitals; that was all very well for many diseases, but for maladies of the nervous system it was just as well to have a small hospital rather than a large one. These small hospitals should be dotted about in various districts. His Grace did not at all acquiesce in the banishment of pictures for fear of microbes.

The Mayor of Marylebone (Mr. C. J. Elgood) proposed a resolution, in the name of the assembly, wishing prosperity and a useful career to the hospital and giving a pledge of support. The Mayor's acquaintance with the hospital was only of recent date, but he said he was glad it had been situated in a great thoroughfare, where it was always in the public sight. With regard to the rating question, Mr. Elgood stated that the London County Council were placed over the borough councils and made very strict investigations as to the full ratings of buildings. He suggested an appeal to Parliament to exempt hospitals in the same way that elementary schools were exempted.

Sir Henry Burdett, who seconded the resolution, showed the uselessness of any further appeal to Parliament on this point, as the whole matter had been thoroughly thrashed out some years ago when it was decided that all Government buildings were to be rated, it having been found that so large a remission of rates was a danger to the commonwealth. The only solution was that the Assessment Committee in Marylebone should agree only to levy rates on that portion of the building that was not beneficially occupied, which would be that part inhabited by the staff. In these days of empire-building it is not sufficiently borne in mind that the strength of the Empire rests upon the well-being of the individual. In the race to get rich and in the struggle for existence the strain upon the constitutions and frames of men and women was so great that there were increasing numbers of cases of breakdown of the nervous system. Especially were these noticeable among those who worked in the City of London, where, if a man was a hard worker, however much care he took of his own health, the strain was so great that the wise worker insured against a break down. It was the custom now to insure against every form of disease—he himself had been insured for 25 years against paralysis. The one general way in which a man could insure against nervous diseases was to see that adequate provision was made for the study of these diseases, so that medical knowledge and skill should always be available for the worker when he might fall out of the ranks. These hospitals for paralysis and nervous diseases were absolutely necessary. It was a very interesting fact that where we had sufficient hospital accommodation for the treatment of a certain disease, the number of cases under treatment grew steadily less. In illustration of this point Sir Henry quoted some figures which showed—taking all the patients treated at the medical institutions of London—that, whereas consumption patients had decreased 6 per cent. during the eight years 1893 to 1901, and cases of patients suffering from skin diseases had decreased 10 per cent., the number of those suffering from nervous diseases showed an increase of 133 per cent. It is quite essential to the well-being of Londoners especially that this hospital for epilepsy and paralysis should obtain the £5,000 of income needed for carrying on the work and £12,000 for the building of the new wing without delay.

The resolution was briefly responded to by Mr. Charles Drummond, the treasurer, after which the Royal party proceeded on a tour of inspection of the building, in which they took great interest and expressed their satisfaction.

The three front wards on each floor open upon a broad balcony, on to which beds and couches can be moved, whilst at the back the patients have access on the first floor to a roof-garden over the out-patient department. The hospital is furnished with a hydraulic lift, large enough for a bed, a patient, and two attendants. A separate electric current from a battery in the basement is conveyed to all the wards and consulting-rooms for medical purposes.

